# Suppressive effects of RXR agonist PA024 on adrenal *CYP11B2* expression, aldosterone secretion and blood pressure

**DOI:** 10.1371/journal.pone.0181055

**Published:** 2017-08-11

**Authors:** Dai Suzuki, Akiko Saito-Hakoda, Ryo Ito, Kyoko Shimizu, Rehana Parvin, Hiroki Shimada, Erika Noro, Susumu Suzuki, Ikuma Fujiwara, Hiroyuki Kagechika, William E. Rainey, Shigeo Kure, Sadayoshi Ito, Atsushi Yokoyama, Akira Sugawara

**Affiliations:** 1 Department of Pediatrics, Tohoku University Graduate School of Medicine, Sendai, Miyagi, Japan; 2 Department of Molecular Endocrinology, Tohoku University Graduate School of Medicine, Sendai, Miyagi, Japan; 3 Institute of Biomaterials and Bioengineering, Tokyo Medical and Dental University, Chiyoda-ku, Tokyo, Japan; 4 Department of Molecular and Integrative Physiology, University of Michigan Medical School, Ann Arbor, Michigan, United States of America; 5 Division of Nephrology, Endocrinology and Vascular Medicine, Tohoku University Graduate School of Medicine, Sendai, Miyagi, Japan; Hokkaido Daigaku, JAPAN

## Abstract

The effects of retinoids on adrenal aldosterone synthase gene (*CYP11B2*) expression and aldosterone secretion are still unknown. We therefore examined the effects of nuclear retinoid X receptor (RXR) pan-agonist PA024 on *CYP11B2* expression, aldosterone secretion and blood pressure, to elucidate its potential as a novel anti-hypertensive drug. We demonstrated that PA024 significantly suppressed angiotensin II (Ang II)-induced *CYP11B2* mRNA expression, promoter activity and aldosterone secretion in human adrenocortical H295R cells. Human *CYP11B2* promoter functional analyses using its deletion and point mutants indicated that the suppression of *CYP11B2* promoter activity by PA024 was in the region from -1521 (full length) to -106 including the NBRE-1 and the Ad5 elements, and the Ad5 element may be mainly involved in the PA024-mediated suppression. PA024 also significantly suppressed the Ang II-induced mRNA expression of transcription factors NURR1 and NGFIB that bind to and activate the Ad5 element. NURR1 overexpression demonstrated that the decrease of NURR1 expression may contribute to the PA024-mediated suppression of *CYP11B2* transcription. PA024 also suppressed the Ang II-induced mRNA expression of *StAR*, *HSD3β2* and *CYP21A2*, a steroidogenic enzyme group involved in aldosterone biosynthesis. Additionally, the PA024-mediated *CYP11B2* transcription suppression was shown to be exerted via RXRα. Moreover, the combination of PPARγ agonist pioglitazone and PA024 caused synergistic suppressive effects on *CYP11B2* mRNA expression. Finally, PA024 treatment significantly lowered both the systolic and diastolic blood pressure in Tsukuba hypertensive mice (hRN8-12 x hAG2-5). Thus, RXR pan-agonist PA024 may be a candidate anti-hypertensive drugs that acts via the suppression of aldosterone synthesis and secretion.

## Introduction

Hypertension has long been recognized as a major risk factor for cardiovascular disease, stroke, and chronic kidney disease [1]. In 2010, the number of patients with hypertension was reported to be 31.1% of the world’s adult population (an estimated 1.39 billion people) [2], and the prevalence has been reported to be increasing, especially in low- and middle-income countries since 2000 [2]. Blood pressure control is very important to reduce complications and the mortality risk. In many cases, however, sufficient hypotensive effects are not obtained from lifestyle modification or popular anti-hypertensive drugs such as angiotensin converting enzyme inhibitors, angiotensin II (Ang II) receptor blockers (ARBs), thiazide-diuretics and calcium channel blockers. In particular, approximately 20–30% of hypertensive patients are estimated to be ‘‘resistant hypertension” in spite of the concurrent use of more than 3 anti-hypertensive agents [3]. In order to treat the growing number of patients with ‘‘resistant hypertension,” the development of novel antihypertensive drugs are needed.

Retinoids, which are natural and synthetic vitamin A derivatives, regulate a wide range of biological processes including development, differentiation, proliferation, and apoptosis. Retinoids exert their effects through retinoic acid receptors (RARα, β, γ) and retinoid X receptors (RXRα, β, γ), which are members of the nuclear steroid/thyroid hormone receptor superfamily [4]. Whereas RARs form a heterodimer with RXRs alone, RXRs form a homodimer or a heterodimer with other nuclear receptors including peroxisome proliferator-activated receptors (PPARs) and liver X receptors (LXRs) as well as RARs, and regulate various transcriptional activities by binding to the specific DNA response element of the target gene [5].

Various RXR selective agonists, which are also called rexinoids, have recently been developed, and some of them have shown anti-tumor effects both *in vivo* and *in vitro* [4,6–9]. Indeed, bexarotene, a novel oral selective RXR agonist, has already been approved for the treatment of refractory cutaneous T-cell lymphomas (CTCLs) and non-small cell lung cancer (NSCLC) in human [9,10]. We recently demonstrated that both synthetic RXR pan-agonist HX630 and PA024 induced apoptosis and inhibited proliferation in murine pituitary corticotroph tumor AtT20 cells. We also demonstrated that HX630 inhibited tumor growth in *vivo* and decreased pro-opiomelanocortin gene (*Pomc*) mRNA expression and in *vitro*. Thus, we provide new evidence that RXR agonists, especially HX630 could be new therapeutic candidates for Cushing’s disease [11].

Aldosterone, secretion of which is mainly regulated by Ang II and serum potassium, is one of the most important hormone in the regulation of the systemic blood pressure through the absorption of sodium and water. Aldosterone production is regulated tightly by selective expression of aldosterone synthase (CYP11B2) at the regulatory step in the adrenal outer-most zone, the zona glomerulosa [12,13]. We have previously shown that the PPARγ agonist pioglitazone inhibited both Ang II- and potassium-mediated aldosterone synthase gene (*CYP11B2*) transcriptional activation via Ca^2+^/calmodulin-dependent kinase (Ca^2+^-CaM-CaMK) inhibition in H295R cells derived from human adrenocortical carcinoma [14]. On the other hand, there has been no report showing the effects of RXR, which is a heterodimer partner of PPAR, on *CYP11B2* expression and aldosterone secretion in adrenocortical cells. The objectives of this study are to examine the effects of the RXR pan-agonist PA024 on *CYP11B2* expression, aldosterone secretion, and blood pressure, and to elucidate its molecular mechanisms for the future innovation of novel anti-hypertensive drugs.

## Materials and methods

### Reagents

Human angiotensin II (Ang II) was purchased from Sigma (St. Louis, MO). Ang II was dissolved in PBS and 0.1% bovine serum albumin (BSA) at 100 μmol/L and stored at -80°C. These stocks were diluted with medium to 100 nmol/L immediately before each experiment. RXR pan-agonist PA024 was previously described [15,16]. The PPARγ agonist pioglitazone was purchased from Alexis Biochemicals (Farmingdale, NY, USA). Each drug was dissolved in DMSO at 10 mmol/L and stored at -20°C. These stocks were diluted with medium to the desired concentration immediately before each experiment, keeping the final concentration of DMSO at 0.1%.

### Plasmids

The subcloned chimeric constructs containing the human *CYP11B2* genomic DNA and luciferase cDNA (pGL3-basic, Promega, Madison, WI) [14,17] were used for the transient transfection studies: -1521/+2-luc (harboring the *CYP11B2* 5’-flanking region from -1521 to +2 relative to the transcription start site upstream of the luciferase cDNA in pGL3-basic); -747/+2-luc; -135/+2-luc; -106/+2-luc; -65/+2-luc. β-Galactosidase control plasmid in pCMV (pCMV-β-gal) was purchased from Clontech (Mountain View, CA). Murine nerve growth factor-induced clone B (NGFIB) and Nur-related factor 1 (NURR1) cDNA were cloned by PCR from murine pituitary AtT20 cell RNA and subcloned into the pcDNA3 expression vector (Invitrogen, Carlsbad, CA) (NGFIB-pcDNA3 and NURR1-pcDNA3). Murine RXRα and RXRβ cDNA previously subcloned into pcDNA1/Amp expression vector (Invitrogen) (RXRα-pcDNA1/Amp, RXRβ-pcDNA1/Amp) [18] were also used. Several vectors were mutated using a QuikChange site-directed mutagenesis kit (Stratagene, La Jolla, CA); NBRE-1 element in -1521/+2-luc from 5’-AAAGGCTA-3’ (-766/-759) to 5’-gAAttCTA-3’ (-1521/+2-luc-NBRE-1-mut); Ad5 element in -1521/+2-luc from 5’-GACCTT-3’ (-129/-114) to 5’-GAtaTc-3’ (-1521/+2-luc-Ad5-mut); Ad1/CRE element in -1521/+2-luc from 5’TGACGTGA-3’ (-71/-64) to 5’-gGtaccGA-3’ (-1521/+2-luc-Ad1/CRE-mut) [14,19].

### Cell culture

NCI-H295R (H295R) human adrenocortical tumor cells purchased from ATCC (American Type Culture Collection) were grown with 1:1 mixture of DMEM and Ham’s F12 medium supplemented with 10% fetal bovine serum (FBS), insulin-transferrin-selenium-G supplements (Invitrogen), 1.25 mg/ml BSA (Sigma), 5.35 mg/ml linoleic acid (Sigma), 100 U/ml penicillin, and 100 mg/ml streptomycin. Cells were cultured in a humidified incubator at 37°C with 5% CO_2_.

### Proliferation assay

The cell numbers were counted using a Cell Counting Kit-8 (Dojindo, Kumamoto, Japan) according to the manufacturer’s instructions. Briefly, H295R cells (5×10^3^ cells/well) seeded in 96-well plates were incubated in 100 μl regular media for several days. The cells were then re-fed with DMEM supplemented with 1% stripped FBS media containing appropriate concentrations of PA024. After incubation for 48 h, 10 μl of assay reagent were added onto each well and the plate was incubated for 4 h at 37°C, 5% CO_2_. The generation of colored formazan product, which was bio-reduced from tetrazolium compound in metabolically active cells, was assessed optically by measuring the absorbance at 450 nm (reference 600 nm) using a microplate reader. Results are expressed as percentages of control.

### Apoptosis assay

Cell apoptosis was estimated using a Homogeneous Caspases Assay, (fluorimetric) kit (Roche, Mannheim, Germany) according to the manufacturer’s instructions. Briefly, H295R cells (5×10^3^ cells/well) seeded in 96-well plates were incubated in 100 μl regular media for several days. The cells were then re-fed with DMEM supplemented with 1% stripped FBS media containing appropriate concentrations of PA024. After incubation for 48 h, 100 μl of substrate solution was added onto each well, and the plates were incubated for 2 h at 37°C, 5% CO_2_. Activated caspases (2, 3, 6, 7, 8, 9, and 10) from apoptotic cells were detected by measuring the fluorescence using a microplate fluorescence reader with a 485 nm excitation filter and 535 nm emission filter. Results are expressed as percentages of control.

### RNA isolation and quantitative real-time PCR

When H295R cells were grown to 70% confluence in regular medium in 24-multiwell plates, they were incubated either without or with PA024 at appropriate concentrations in DMEM supplemented with 1% stripped FBS media for 24 h. Then the cells were co-treated with 100 nmol/L Ang II for last 6 h. In the overexpression experiments, each expression vector was transfected for 48 h before treatment with PA024. The cells were then lysed and their total RNAs were isolated using ISOGEN (Nippon Gene, Tokyo, Japan) according to the manufacturer’s instructions. Samples were eluted in RNase-free water (50 μl). The RNA was quantified by a Nanodrop 2000 (Thermo Scientific, Waltham, MA). Reverse transcription-polymerase chain reaction (PCR) was performed using Primer script RT master Mix (Takara Bio). Total RNA was converted to cDNA at 37°C for 15 min with reverse transcriptase, oligo dT primer and random 6 mers. Thereafter, Reverse transcription mixtures were subjected to quantitative real-time PCR (95°C, 3 min for 1 cycle; 95°C, 15 s; 60°C, 10 s; 72°C, 20 s for 40 cycles for *HSD3β1* and 95°C, 3 min for 1 cycle; 95°C, 15 s; 60°C, 10 s; 72°C, 20 s for 40 cycles for the others) with iQ Supermix (for *CYP11B1*, *CYP11B2*, *CYP17A1*, *HSD3β1* and *HSD3β2*) or iQ SYBR green Supermix (for others) (Bio-Rad, Hercules, CA) using a DNA Engine thermal cycler attached to a Chromo4 detector (Bio-Rad). The signals of the samples of interest were then quantified from the standard curve, and all obtained data were normalized by β-actin in the human primer and mouse 18S rRNA in the mouse primer. To confirm the amplification specificity, the PCR products from each primer pair were subjected to a melting curve analysis with SYBR green. Results are expressed as percentages of each control. The sequences of the primers and TaqMan probe are shown in Table 1.

**Table 1:** Primer and TaqMan probe sequences for real time PCR.

**Gene**	**Forward primer (5’-3’)**	**Reverse primer (3’-5’)**	**Product Size (bp)**
CYP11B2	GGCAGAGGCAGAGATGCTG	CTTGAGTTAGTGTCTCCACCAGGA	71
STAR	GCATCCTTAGCAACCAAGAG	TCACTTTGTCCCCATTGTCC	63
CYP11A1	TTCCGCTTTGCCTTTGAGTC	TGGCATCAATGAATCGCTGG	93
HSD3β1	AGAAGAGCCTCTGGAAAACACATG	TAAGGCACAAGTGTACAGGGTGC	127
HSD3β2	GCGGCTAATGGGTGGAATCTA	CATTGTTGTTCAGGGCCTCAT	117
CYP21A2	AGACTACTCCCTGCTCTGGA	CTCATGCGCTCACAGAACTC	123
CYP17A1	CAGAATGTGGGTTTCAGCCG	CTCACCGATGCTGGAGTCAA	150
CYP11B1	GGCAGAGGCAGAGATGCTG	TCTTGGGTTAGTGTCTCCACCTG	72
NURR1	AGAGAAGATCCCTGGCTTCG	CAAGACCACCCCATTGCAAAA	148
NGFIB	CCTGGAGCTCTTCATCCTCC	TGTCAATCCAGTCCCCGAAG	125
β-actin	CCAACCGCGAGAAGATGACC	CCAGAGGCGTACAGGGATAG	97
Mouse RXRα	GGCTTCGGGACTGGTAGCC	GCGGCTTGATATCCTCAGTG	73
**Gene**	**TaqMan probe**		
CYP11B2	[6-FAM]CTGCACCACGTGCTGAAGCACT[TAMRA6-FAM]		
CYP11B1	[6-FAM]TGCTGCACCATGTGCTGAAACACCT[TAMRA6-FAM]		
HSD3β1	[6-FAM]CCATACCCACACAGC[NFQ-MGB]		
HSD3β2	[6-FAM]TGATACCTTGTACACTTGTGC[TAMRA6-FAM]		
CYP17A1	[6-FAM]TCAGTGACCGTAACCGTCTC[TAMRA6-FAM]		

STAR, steroidogenic acute regulatory protein; HSD3β1/2, hydroxy-delta-5-steroid dehydrogenase, 3 beta- and steroid delta-isomerase 1/2; NURR1, nuclear receptor related 1 protein; NGFIB, nerve growth factor-induce clone B; Mouse RXRα, mouse retinoid X receptor α

### Transient transfection and luciferase assay

When H295R cells were grown to 60% confluence in 24-multiwell plates, they were transiently transfected with 200 ng luciferase reporter plasmids and 100 ng pCMV-β-gal using Lipofectamine LTX and Plus reagent (Invitrogen) for 24 h according to the manufacturer’s instructions. In the overexpression experiments, 100–300 ng of each expression vector including NGFIB-pcDNA3, NURR1-pcDNA3, RXRα-pcDNA1/Amp or RXRβ-pcDNA1/Amp were also transfected. The media were changed to DMEM supplemented with 1% resin and charcoal-treated (stripped) FBS, and the cells were incubated either without or with PA024 at appropriate concentrations for 24 h. Then the cells were co-treated with 100 nmol/L Ang II for last 6 h. After appropriated treatments, they were washed with PBS, and the cell extracts were prepared using Glo Lysis Buffer (Promega). Luciferase activity was measured using Bright-Glo reagents (Promega), and β-galactosidase activity was simultaneously measured. Data were normalized by β-galactosidase activity.

### Measurement of aldosterone and cortisol concentration

When H295R cells were grown to 70% confluence in 24-multiwell plates, they were incubated either without or with 10 μmol/L PA024 for 24 h in DMEM supplemented with 1% stripped FBS. Next, the media were freshly changed, and the cells were incubated again either without or with 10 μmol/L PA024 and 100 nmol/L Ang II for 24 h. The aldosterone and cortisol concentrations of the media were thereafter measured by Aldosterone EIA kit (Cayman Chemical, Ann Arbor, MI, USA) and Cortisol ELISA kit (Cayman Chemical) according to the manufacturer’s instructions, respectively. The obtained data were normalized by the protein concentrations measured by Protein Assay Kit (Bio-Rad).

### Western blot analyses

When H295R cells were grown to 70% confluence in regular medium in 6 cm dishes, they were incubated in the presence (10 μmol/L) or the absence of PA024 in DMEM supplemented with 1% stripped FBS media for 24 h and were co-treated with 100 nmol/L Ang II for the last 6 h. The cells were then harvested and lysed with TNE buffer (20 mmol/L Tris-HCl, 137 mmol/L NaCl, 2 mmol/L EDTA, 1% NP-40, Protease Inhibitor Cocktail Set III (Calbiochem), pH 7.9). Thereafter, 10 μg of extracted protein were electrophoresed on a SDS-polyacrylamide gel and transferred onto PVDF membrane. For the detection of NURR1, the membrane was blocked with 1% BSA for overnight at 4°C and probed with the primary antibody for NURR1 (sc-991, Santa Cruz Biotechnology, Dallas, TX) (diluted at 1:500) for 3 h at room temperature, and was thereafter incubated with anti-rabbit IgG, horseradish peroxidase (HRP) linked whole antibody from donkey (NA934V, GE Healthcare Life Sciences, Pittsburgh, PA) (1:5000) for 1 h at room temperature. For the detection of actin, the membrane was blocked with 1% BSA for overnight at 4°C and probed with the primary antibody for actin (sc-1616, Santa Cruz Biotechnology) (diluted at 1:500) for 3 h at room temperature, and was thereafter incubated with anti-goat IgG, HRP pre-absorbed from donkey (ab97120) (1:5000) for 1 h at room temperature. Thereafter, the membranes were washed and were visualized using ECL (Bio-Rad). Densitometric analyses of the membranes were performed using Image J.

### Small interfering RNA

Small interfering RNAs (siRNAs) for RXRα (SI00046144) and negative control siRNA (1027280) were obtained from Qiagen (Hilden, Germany). H295R cells grown to 50% confluence in 24-multiwell plates were transiently transfected with 20 pmol siRNAs using Lipofectamine 2000 reagent (Invitrogen) for 48 h according to the manufacturer's instructions. The cells were incubated either without or with 10 μmol/L PA024 for 24 h. Then the cells were treated with 100 nmol/L Ang II for 6 h. Thereafter they were used for quantitative RT-PCR.

### Animal experiments

Tsukuba hypertensive mice (THM), which are transgenic mice carrying human renin and human angiotensinogen genes kindly provided by Dr. Akiyoshi Fukamizu (Tsukuba University) and Riken BRC were used [20]. THM present excessive Ang II production and chronic hypertension [20]. The mice were housed in temperature- and humidity-controlled cages with a 12-hour/12-hour light/dark cycle, and given standard chow diet and tap water ad libitum. Nine-week-old male THM were divided into 2 groups at random (each n = 5) and then administered either vehicle alone (corn oil) or PA024 (10 mg/kg/day) dissolved with corn oil intraperitoneally 3 times a week for 7 weeks. Their body weight (BW), heart rate (HR), systolic blood pressure (SBP) and diastolic blood pressure (DBP) were monitored once a week throughout the treatment. BW was measured by an electronic scale (FY-3000, A&D Company, Tokyo). HR and BP were measured 8 to 12 hours after injection between 14:00–18:00 h by a programmable sphygmomanometer (BP-98A-L, Softron, Tokyo) using the tail-cuff method [20]. Unanesthetized mice were introduced into a small holder mounted on a thermostatically controlled warming plate and maintained at 37°C during the measurement.

All animal experiments were performed in accordance with the institutional regulations for animal care of Tohoku University Graduate School of Medicine. The protocol was approved by the Committee on the Ethics of Animal Experiments of the Tohoku University. Animals were sacrificed by isoflurane anesthesia, and all efforts were made to minimize suffering.

### Measurement of intracellular calcium

H295R cells (4×10^4^ cells/well) seeded in 96-well plates were incubated in 100 μl regular media for several days. Then they were incubated either without or with PA024 at appropriate concentrations in DMEM supplemented with 1% stripped FBS media for 24 h. Thereafter, the cells were loaded with Fluo4-AM (Dojindo, Kumamoto, Japan; 5 mg/ml) in the presence of 1.25 mmol/l probenecid (Dojindo) and 0.04% Pluronic F-12 (Dojindo) for 1 h. They were then washed with PBS, and the recording medium containing 1.25 mmol/l probenecid, and Ang II (100 nmol/L) was added to the media. The change of intracellular calcium was determined by fluorescent intensity (excitation at 485 nm, emission at 535 nm).

### Statistical analysis

All data are presented as mean ± SEM. Statistical analyses were performed with ANOVA followed by Fisher’s least significant difference post hoc test.

## Results

### Effects of PA024 on cell proliferation and apoptosis in H295R cells

**Fig 1:**
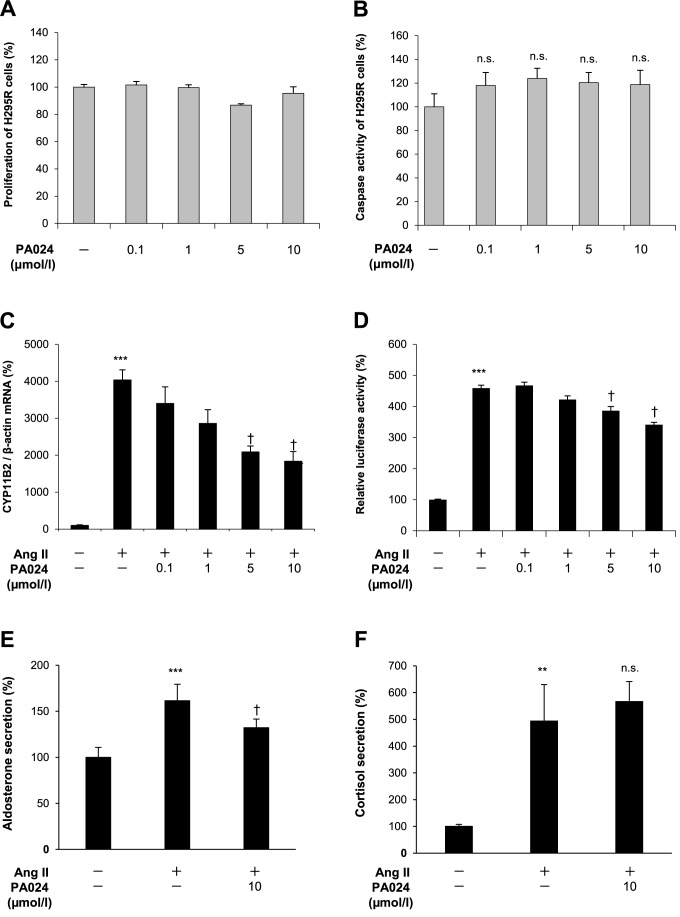
Effects of RXR pan-agonist PA024 on cell proliferation/apoptosis, *CYP11B2* mRNA expression/promoter activity, and aldosterone secretion in H295R cells. Effects of PA024 on proliferation and apoptosis are shown in (A) and (B), respectively. H295R cells were incubated for 48 h either in the presence (100 nmol/L, 1 μmol/L, 5 μmol/L, 10 μmol/L) or absence (control) of PA024, before each assay. Results are expressed as percentages of each control. Data represent mean ± S.E.M. (n = 6). n.s.: not significant versus control. (C), total RNAs extracted from the cells treated with PA024 (the indicated concentration, 24 h) and Ang II (100 nmol/L, 6 h) were examined for *CYP11B2* mRNA expression by quantitative real-time PCR. (D), H295R cells transiently transfected for 24 h with *CYP11B2*-luc (-1521 to +2-luc) and pCMV-β-gal were treated with PA024 (indicated concentrations, 24 h) and Ang II (100 nmol/L, 6 h), before the luciferase assay. Data represent mean ± S.E.M. (n = 4), percent of control. (E) and (F), supernatants obtained from the cells treated with PA024 (10 μmol/L, 48 h) and Ang II (100 nmol/L, 24 h) were examined for aldosterone and cortisol secretion by EIA, respectively. (E), the experiments were performed twice, and each data set (n = 4) was pooled and shown as one data set (n = 8). Data represent mean ± S.E.M. (n = 8), percent of control. (F), data represent mean ± S.E.M. (n = 4), percent of control. ***P<0.001, **P<0.01 vs. control, ^†^*P*<0.05, n.s.: not significant vs. Ang II.

We examined the effects of RXR pan-agonist PA024 on H295R cells proliferation using a WST-8 assay after incubation with various concentrations of PA024 for 48 h. As shown in Fig 1A, PA024 did not affect the proliferation of H295R cells. We next examined the effects of PA024 on the apoptosis of H295R cells by activated caspase assay. There was no significant difference in the caspase activity between each group (Fig 1B). These data suggest that PA024 treatment is not cytotoxic and does not induce apoptosis.

### Effects of PA024 on *CYP11B2* mRNA expression, promoter activity and aldosterone secretion in H295R cells

We examined the effects of PA024 on *CYP11B2* mRNA expression and promoter activity in H295R cells. Ang II treatment significantly increased *CYP11B2* mRNA expression and promoter activity in H295R cells. On the other hand, co-treatment with PA024 significantly suppressed Ang II-induced *CYP11B2* mRNA expression and promoter activity in a dose-dependent manner (Fig 1C and 1D), respectively. We next examined the effects of PA024 on aldosterone secretion from H295R cells to the supernatants. Ang II treatment induced aldosterone secretion as well as mRNA expression and transcriptional activity in H295R cells, but 10 μmol/L PA024 significantly suppressed Ang II-induced aldosterone secretion (Fig 1E). These data suggest that PA024 negatively regulates Ang II-mediated *CYP11B2* transcription, resulting in the suppression of *CYP11B2* mRNA expression and aldosterone secretion in H295R cells.

### Effects of PA024 on the *CYP11B2* promoter deletion mutants and point mutants

**Fig 2:**
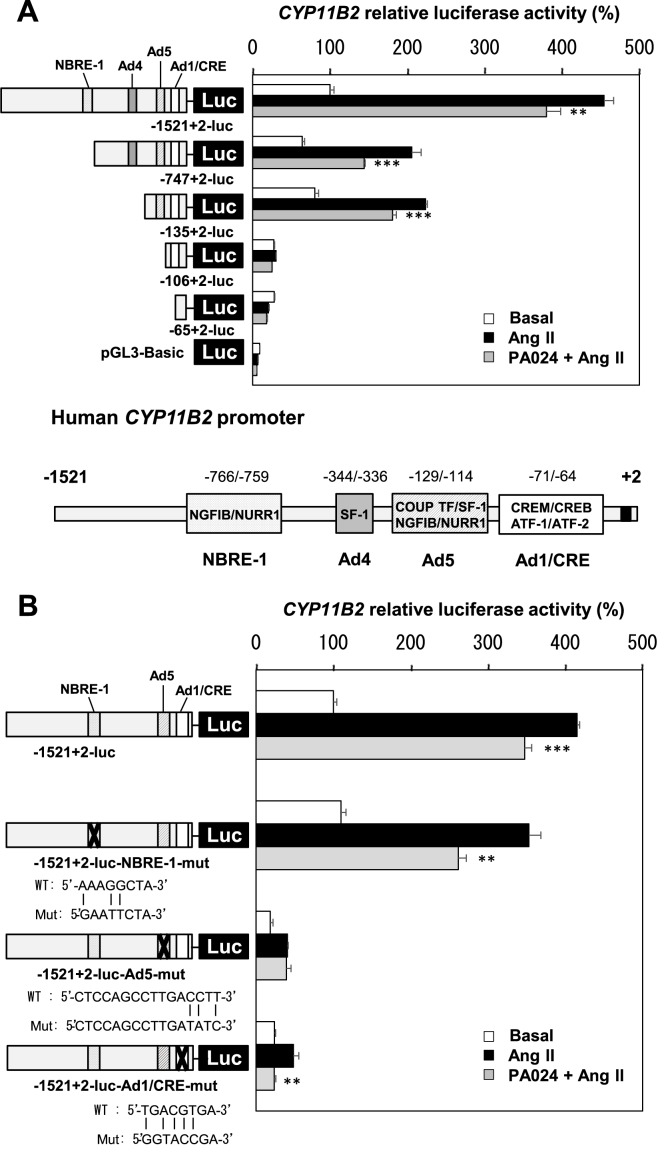
Effects of PA024 on the *CYP11B2* promoter deletion mutants and point mutants. (A), effect of PA024 on the *CYP11B2* promoter deletion mutants. Either -1521/+2-luc, -747/+2-luc, -135/+2-luc, -106/+2-luc, -65/+2-luc, or pGL3-Basic (control plasmid) was transiently transfected for 48 h with pCMV-β-gal into H295R cells, and the cells were thereafter treated with PA024 (10 μmol/L, 24 h) and Ang II (100 nmol/L, 6 h). Data represent mean ± S.E.M. (n = 4), percent of -1521/+2-luc control. (B), effect of PA024 on the *CYP11B2* promoter point mutants. Either -1521/+2-luc, -1521/+2-luc-NBRE-1-mut, -1521/+2-luc-Ad5-mut, or -1521/+2-luc-Ad1/CRE-mut was transiently transfected for 48 h with pCMV-β-gal into H295R cells, and the cells were thereafter treated with PA024 (10 μmol/L, 24 h) and Ang II (100 nmol/L, 6 h). Data represent mean ± S.E.M. (n = 4), percent of -1521/+2-luc control. ***P<0.001, **P<0.01 vs. Ang II.

To identify the elements in the *CYP11B2* promoter involved in the suppression of transcriptional activity by PA024, we first examined the promoter activity of the *CYP11B2* 5’-flanking region deletion mutants. The PA024-mediated suppression of the *CYP11B2* promoter activity observed in the 5’-flanking region from -1521/+2 relative to the transcription start site gradually diminished in parallel with gradual deletions from -1521 to -106 including NBRE-1 (-766/-759), Ad4 (-344/-336) and Ad5 (-129/-114) elements (Fig 2A). There exist three important *cis*-elements for Ang II-induced *CYP11B2* expression in the *CYP11B2* promoter: NBRE-1, Ad5 and Ad1/CRE (-71/-64) [12,13,19]. Therefore we next examined the promoter activity of the *CYP11B2* 5’-flanking legion point mutants. Although Ad5 mutation affected the suppression by PA024, NBRE-1 and Ad1 mutations did not affect the suppression (Fig 2B). These data suggested the possibility that the Ad5 element is involved in the PA024-mediated suppression of the *CYP11B2* promoter activity.

### Possible involvement of NURR1 on the PA024-mediated suppression of *CYP11B2* promoter activity via the Ad5 element

**Fig 3:**
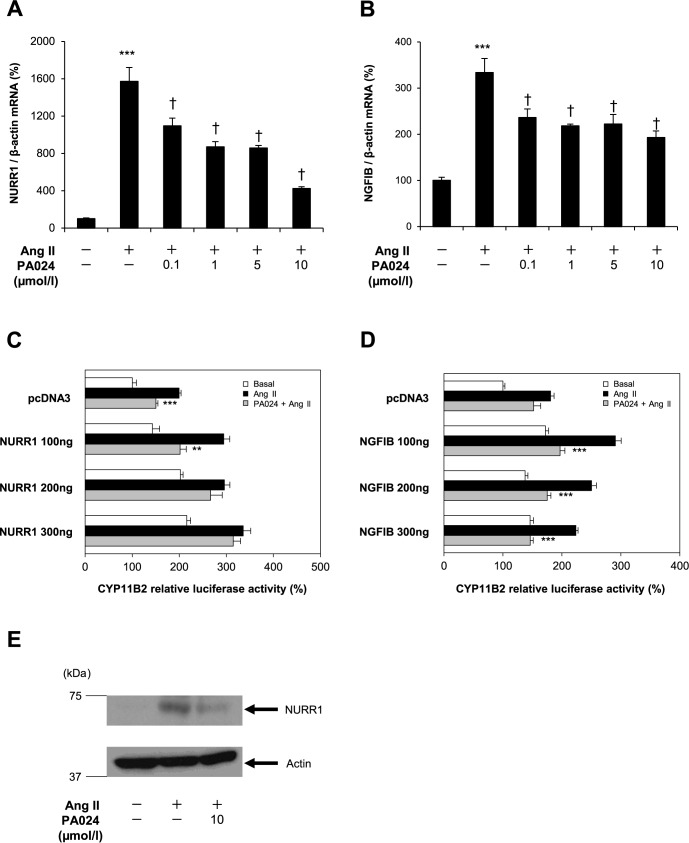
Possible involvement of NURR1 in the PA024-mediated suppression of *CYP11B2* promoter activity via binding to the Ad5 element. (A) and (B), dose-response analyses of the NURR1 and NGFIB mRNA expression. H295R cells were treated with PA024 (indicated concentrations, 24h) and Ang II (100 nmol/L, 6 h). Data represent mean ± S.E.M. (n = 4), percent of control. ***P<0.001 vs. control, ^†^*P*<0.05 vs. Ang II. (C) and (D), effect of NURR1 or NGFIB overexpression on the *CYP11B2* promoter activity. H295R cells transiently transfected with NURR1-pcDNA3, NGFIB-pcDNA3 or pcDNA3 (Mock) for 48 h were treated with PA024 (10 μmol/L, 24 h) and Ang II (100 nmol/L, 6 h), respectively. Data represent mean ± S.E.M. (n = 4), percent of pcDNA control. ***P<0.001, **P<0.01 vs. Ang II. (E), effect of PA024 on NURR1 protein expression. H295R cells were treated with PA024 (10 μmol/L, 24 h) and Ang II (100 nmol/L, 6 h). Upper panel, NURR1 protein expression indicated by arrow. Lower panel, actin protein expression indicated by arrow.

There are two transcription factors that act the Ad5 element in the *CYP11B2* promoter: NURR1 and NGFIB [12,13,19]. Therefore, we first examined the effects of PA024 on the NURR1 and NGFIB mRNA expression levels in H295R cells. PA024 significantly suppressed the Ang II-induced mRNA expression of NURR1 and NGFIB in a dose-dependent manner (Fig 3A and 3B). Next, we examined the effects of NURR1 and NGFIB overexpression on the *CYP11B2* promoter activity. NURR1 overexpression rescued the PA024-mediated suppression of the *CYP11B2* promoter activity (Fig 3C), whereas NGFIB overexpression did not (Fig 3D). As shown in Fig 3E, PA024 treatment also significantly decreased Ang II-induced NURR1 protein expression approximately to 0.4-fold by Western blot analyses. These data indicate the decrease of Ad5 activation via NURR1 decrease may contribute to the PA024-mediated suppression of *CYP11B2* transcription.

### Effects of PA024 on mRNA expression of other enzymes involved in aldosterone synthesis

**Fig 4:**
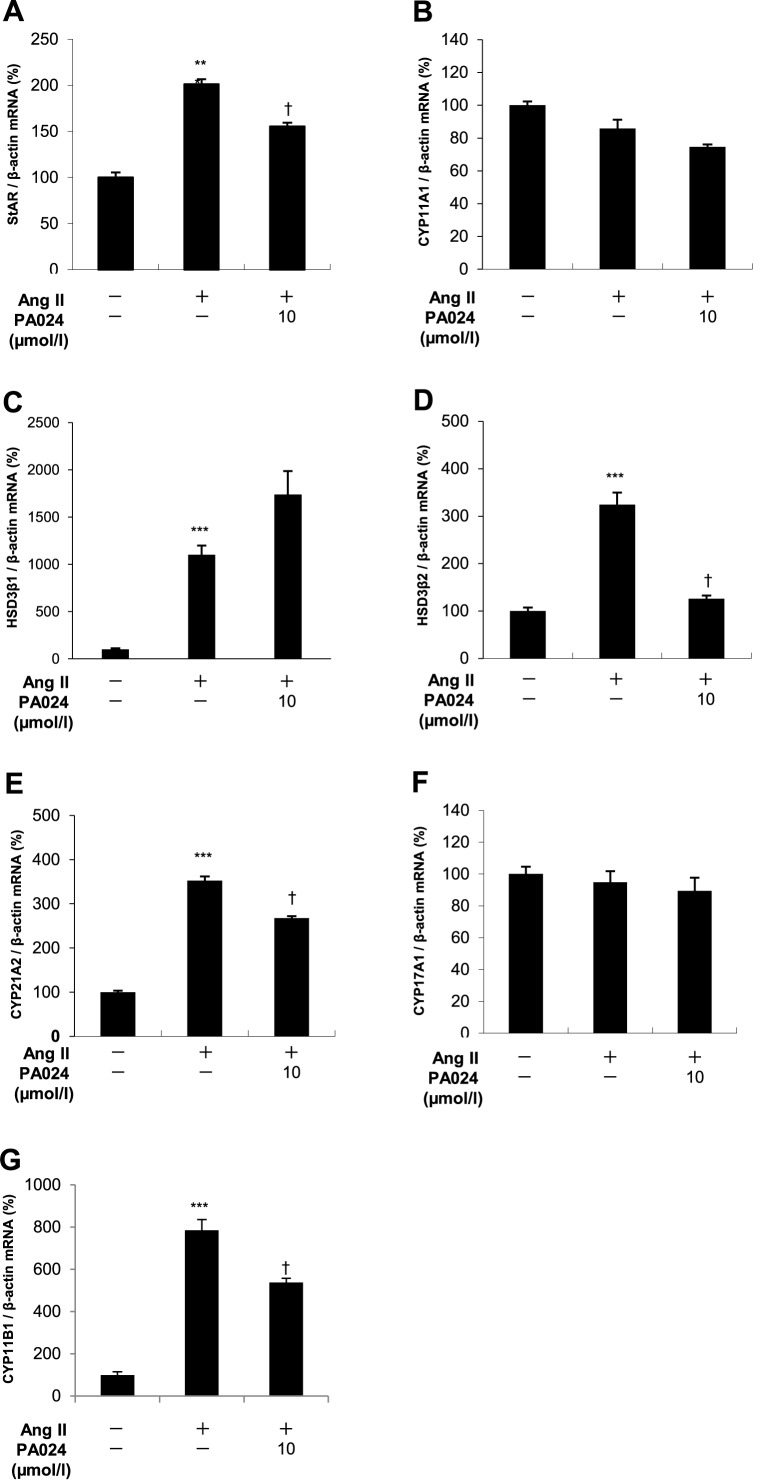
Effects of PA024 on mRNA expression of other enzymes involved in steroid synthesis. (A)-(G), total RNAs extracted from the cells treated with PA024 (10 μmol/L, 24 h) and Ang II (100 nmol/L, 6 h) were examined for mRNA expression of *StAR*, *CYP11A1*, *HSD3β1*, *HSD3β2*, *CYP21A2*, *CYP17A1* and *CYP11B1* by quantitative real-time PCR. Data represent mean ± S.E.M. (n = 4), percent of control, normalized by β-actin mRNA levels. ***P<0.001 vs. control, ^†^*P*<0.05 vs. Ang II.

Aldosterone biosynthesis is regulated by several steroidogenic enzymes group [21]. The expression of steroidogenic acute regulatory (StAR) protein, which is involved in the transport of cholesterol into the mitochondria in the early rate-limiting step of aldosterone biosynthesis, is stimulated by Ang II both *in vitro* [22–24] and *in vivo* [25,26]. Ang II also stimulates the expressions of steroidogenic enzymes, which are involved in subsequent steps of aldosterone biosynthesis, including cholesterol side-chain cleavage (SCC or *CYP11A1*) [27], type II 3β-hydroxysteroid dehydrogenase (*HSD3β2*) [28], and *CYP21A2* [29]. Ang II treatment significantly increased mRNA expressions of *StAR*, *HSD3β2* and *CYP21A2* in H295R cells, while 10 μmol/L PA024 significantly suppressed Ang II-induced mRNA expressions of these enzymes (Fig 4A, 4D and 4E). These data suggest that these enzymes may also be involved in the PA024-mediated suppression of aldosterone synthesis.

### Effects of PA024 on *CYP11B1* mRNA expression and cortisol secretion

*CYP11B1* is involved in cortisol synthesis in the zona fasciculata of the adrenal gland, and its gene structure is fairly similar to that of *CYP11B2* [30]. Therefore, we next examined the effects of PA024 on *CYP11B1* mRNA expression in H295R cells and cortisol secretion from H295R cells to the supernatant. Ang II treatment significantly increased *CYP11B1* mRNA expression and cortisol secretion in H295R cells. Co-treatment with 10 μmol/L PA024 significantly suppressed Ang II-induced *CYP11B1* mRNA expression (Fig 4G), wheareas 10 μmol/L PA024 did not suppress Ang II-induced cortisol secretion (Fig 1F). These data suggest that PA024 suppresses Ang II-induced *CYP11B1* mRNA expression, but did not affect the cortisol secretion in H295R cells.

### Involvement of RXRα in the suppressive effect of PA024 on *CYP11B2* mRNA expression and promoter activity

**Fig 5:**
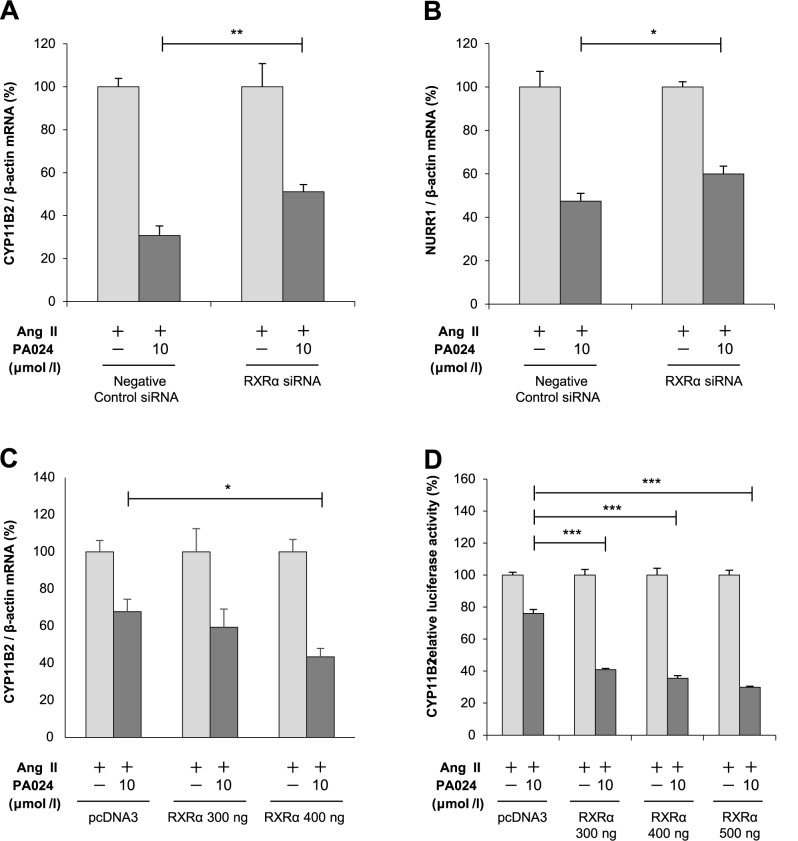
Involvement of RXRα in the suppressive effect of PA024 on *CYP11B2* mRNA expression and promoter activity. (A) and (B), effect of RXRα knockdown by its siRNA on the mRNA expression of *CYP11B2* and NURR1. H295R cells transiently transfected with siRNA (negative control or RXRα) for 48 h were incubated either in the presence (10 μmol/L) or absence (control) of PA024 for 24 h and co-treated with 100 nmol/L Ang II for the last 6 h. Results are expressed as percentages of each Ang II. Data represent mean ± S.E.M. (n = 4). **P<0.01, *P<0.05 vs. negative control siRNA at 10 μmol/L PA024. (C), effect of RXRα overexpression on the *CYP11B2* mRNA expression. H295R cell transiently transfected with RXRα-pcDNA1/Amp (mRXRα) or pcDNA3 (Mock) for 48 h were incubated either in the presence (10 μmol/L) or absence (control) of PA024 for 24 h and co-treated with 100 nmol/L Ang II for the last 6 h. Results are expressed as percentages of each Ang II. Data represent mean ± S.E.M. (n = 4). *P<0.05 vs. pcDNA3 at 10 μmol/L PA024. (D), effect of RXRα overexpression on the *CYP11B2* promoter activity. H295R cell transiently transfected with -1521/+2-luc, pCMV-β-gal, and RXRα-pcDNA1/Amp (mRXRα) or pcDNA3 (Mock) for 48 h were incubated either in the presence (10 μmol/L) or absence (control) of PA024 for 24 h and co-treated with 100 nmol/L Ang II for the last 6 h. Results are expressed as percentages of each Ang II. Data represent mean ± S.E.M. (n = 4). ***P<0.001 vs. pcDNA3 at 10 μmol/L PA024.

We next examined the involvement of RXRα or RXRβ on *CYP11B2* and NURR1 mRNA expression by knockdown using respective small interfering RNA (siRNA). The decrease of endogenous RXRα or RXRβ mRNA expression by each siRNA was confirmed by quantitative real-time PCR (data not shown). Endogenous RXRα knockdown by its siRNA significantly restored the inhibitory effects of PA024 on *CYP11B2* and NURR1 mRNA expression (Fig 5A and 5B). On the other hand, RXRβ knockdown by its siRNA did not affect inhibitory effects of PA024 on *CYP11B2* or NURR1 mRNA expression (data not shown). Moreover, RXRα overexpression significantly augmented the PA024-mediated suppression of *CYP11B2* mRNA expression and promoter activity in a dose-dependent manner (Fig 5C and 5D). The increase of endogenous RXRα mRNA expression by its overexpression was confirmed by quantitative real-time PCR (data not shown). These data suggest that the negative regulation of *CYP11B2* transcription by PA024 is most likely mediated via RXRα.

### Synergistic effects of pioglitazone and PA024 on *CYP11B2* mRNA expression

**Fig 6:**
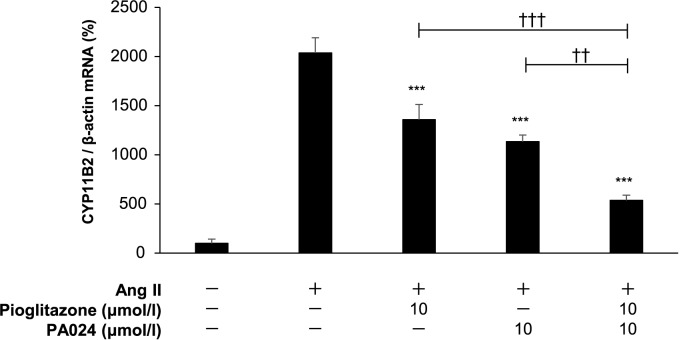
Effects of the combination of pioglitazone and PA024 on *CYP11B2* mRNA expression in H295R cells. Total RNAs extracted from the cells treated with combinations of pioglitazone (10 μmol/L, 24 h), PA024 (10 μmol/L, 24 h), and Ang II (100 nmol/L, 6 h) were examined for *CYP11B2* mRNA expression by quantitative real-time PCR. Data represent mean ± S.E.M. (n = 4), percent of control, normalized by β-actin mRNA levels. ***P<0.001 vs. Ang II, ^†††^*P*<0.001, ^††^*P*<0.01 vs. 10 μmol/L pioglitazone + 10 μmol/L PA024

We next examined the effects of the combination of PPARγ agonist pioglitazone and RXR pan-agonist PA024 on *CYP11B2* mRNA expression. Treatment with either 10 μmol/L pioglitazone or 10 μmol/L PA024 significantly suppressed the Ang II-induced *CYP11B2* mRNA expression to approximately 0.65-fold and 0.55-fold, respectively. Moreover, co-treatment with 10 μmol/L PA024 and 10 μmol/L pioglitazone further suppressed the Ang II-induced *CYP11B2* mRNA expression to approximately 0.25-fold (Fig 6). These data suggest that the combination of PA024 and pioglitazone causes synergistic suppressive effects on *CYP11B2* mRNA expression.

### Effects of PA024 on body weight, heart rate and blood pressure *in vivo*

**Fig 7:**
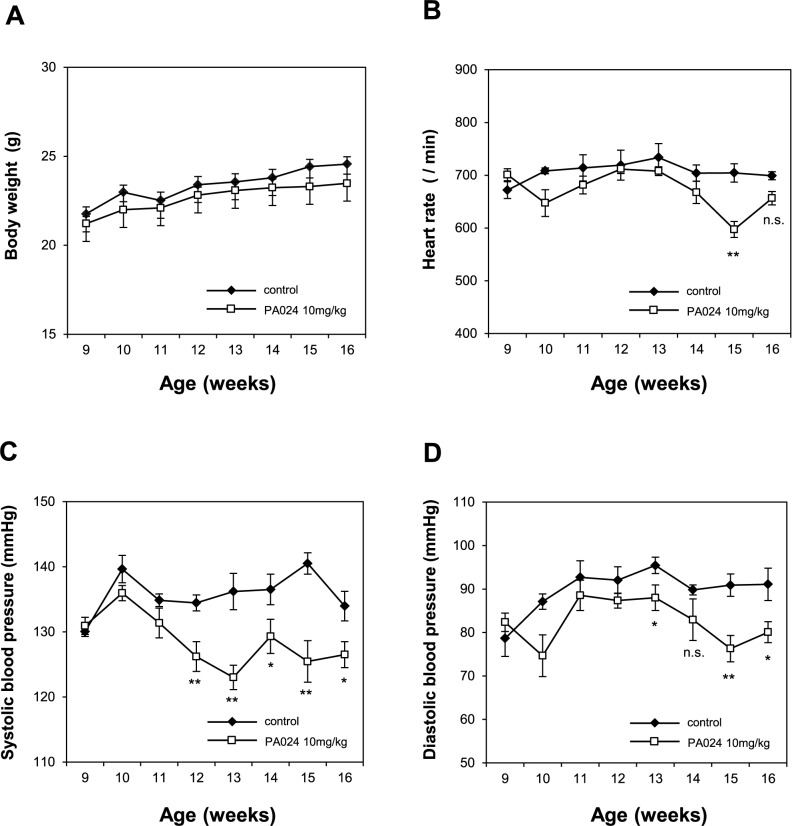
Effects of PA024 on body weight, heart rate, and blood pressure *in vivo*. Nine-week-old Tsukuba hypertensive mice (THM; hRN8-12 x hAG2-5), which present excessive Ang II production and chronic hypertension, were randomized for injection with either vehicle (corn oil) or PA024 (10 mg/kg/day) intraperitoneally 3 times a week for 7 weeks. Body weight (A), heart rate (HR) (B), systolic blood pressure (SBP) (C) and diastolic blood pressure (DBP) (D) were monitored once a week throughout the treatment. Data represent mean ± S.E.M. (n = 5). **P<0.01, *P<0.05, n.s: not significant vs. control mice at each week-old.

Finally, we examined the effects of PA024 *in vivo*. We injected nine-week-old male THM (hRN8-12 x hAG2-5) with PA024 (10 mg/kg/day) or vehicle intraperitoneally 3 times a week for 7 weeks. There was no significant difference in BW change before and after treatment between the control group and the PA024 treated group (Δ2.8±0.12 g versus Δ2.26±0.24 g, *P* = 0.052) (Fig 7A). There was also no significant difference in the HR between the two groups after treatment for 7 weeks (control group: 699±8 /min versus PA024 treated group: 656±13 /min, P = 0.13), although PA024 transiently decreased HR after treatment for 6 weeks (control group: 704±17 /min versus PA024 treated group: 597±15 /min, *P* = 0.0012) (Fig 7B). On the other hand, PA024 significantly decreased both SBP and DBP after a few weeks of treatment (Fig 7C and 7D). Their SBP after treatment for 7 weeks was: 134±2 mmHg in the control group and 127±2 mmHg in the PA024 treated group, respectively. Their DBP after treatment for 7 weeks was: 91±4 mmHg in the control group and 80±2 mmHg in the PA024 treated group, respectively.

## Discussion

There have been few studies showing the relationship between nuclear receptors and *CYP11B2* expression or aldosterone secretion in adrenocortical cells. We previously demonstrated suppressive effects of PPARγ agonist pioglitazone on Ang II-mediated *CYP11B2* expression and aldosterone secretion via Ca^2+^-CaM-CaMK inhibition in H295R cells [14]. On the other hand, Pan et al. reported that pioglitazone promoted *CYP11B2* expression but nevertheless inhibited aldosterone production in Ang II-treated human adrenocortical HAC15 cells, a clone of NCI H295R cells [31]. Therefore, the effect of pioglitazone on *CYP11B2* expression is controversial. Recently, RXR agonists are expected to have therapeutic potential for cancer prevention and treatment. Indeed several RXR agonists (e.g. LG100268, AGN194204, 9-cis UAB30) were found to be effective in suppressing tumor development in multiple carcinogenesis models, including those of breast, skin, pancreas, and prostate [4,6–9]. We also demonstrated that RXR-selective pan-agonists HX630 and PA024 exerted anti-proliferative and pro-apoptotic effects in murine pituitary corticotroph tumor AtT20 cells [11]. Furthermore, we confirmed that HX630 inhibited tumor growth *in vivo*. However, there has been no report showing the effects of RXR agonists on *CYP11B2* expression or aldosterone secretion in adrenocortical cells. In the present study, we demonstrated for the first time regarding the suppressive effects of RXR pan-agonist PA024 on *CYP11B2* expression and aldosterone secretion in H295R cells. Furthermore, we confirmed the hypotensive effect of PA024 *in vivo*.

The regulatory mechanism of human *CYP11B2* expression by Ang II has been elucidated in detail [12,13,19]. Ang II binds to the angiotensin type 1 receptors (AT_1_Rs) and activates Ca^2+^-CaM-CaMK pathway, inducing the expression of NURR1/NGFIB and, furthermore, phosphorylates activating transcription factor (ATF) and cyclic AMP response element binding protein (CREB). NURR1 and NGFIB bind to and activate the NGFIB response element (NBRE-1) and Ad5 element, respectively. ATF and CREB bind to and activate the *CYP11B2* consensus cyclic AMP response element (Ad1/CRE). Therefore, *CYP11B2* gene expression is regulated through these three *cis*-elements (NBRE-1, Ad5 and Ad1/CRE), leading to aldosterone biosynthesis. In the present study, human *CYP11B2* promoter functional analyses using its deletion/point mutants or NURR1 overexpression indicated that the decrease of Ad5 activation via NURR1 decrease may mainly contribute to the PA024-mediated suppression of *CYP11B2* transcription (Figs 2A, 2B and 3C). A previous study reported that mutation of the Ad5 site further reduced both NGFIB- and NURR1-stimulated *CYP11B2* promoter activities compared to the mutation of the NBRE-1 element [19], indicating that the Ad5 element is more important than the NBRE-1 element for *CYP11B2* transcription. Therefore, the Ad5 element may be more predominant than the NBRE-1 element in the PA024-mediated suppression of *CYP11B2* transcription.

**Fig 8:**
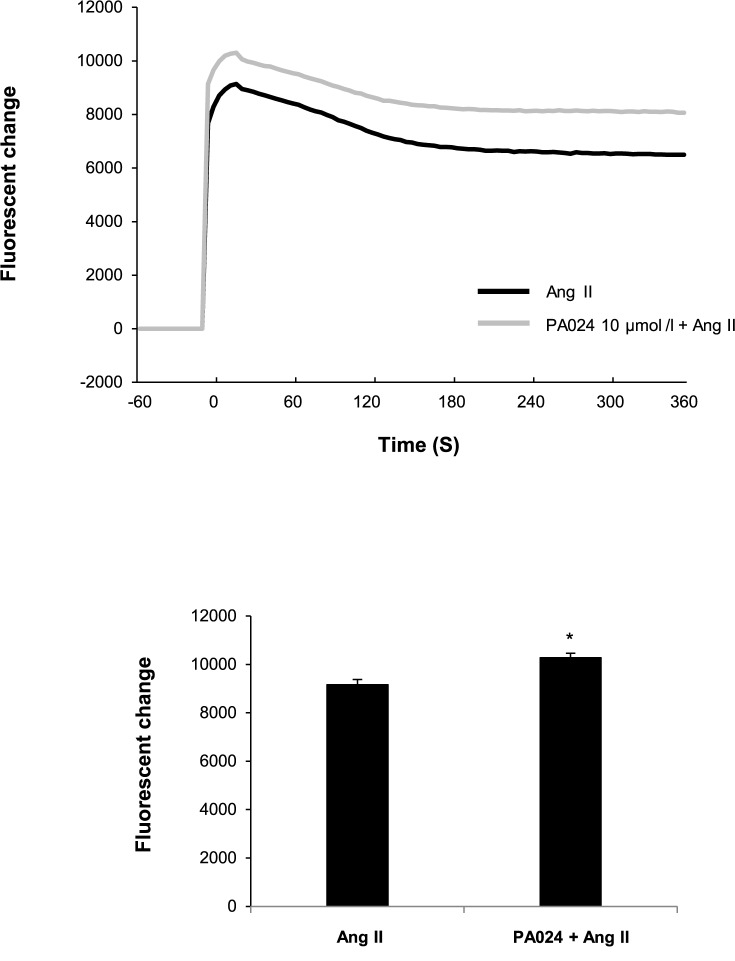
Effects of PA024 on intracellular calcium ion concentration in H295R cells determined by Fluo4-AM. H295R cells were treated with or without PA024 (10 μmol/L, 24 h). After loading with Fluo4-AM, cells were treated with 100 nmol/L Ang II, and the fluorescence change was monitored. Data represent mean (the left panel, n = 6) or mean ± S.E.M. (the right panel, n = 6), fluorescence change from time 0, arbitrary units. *P<0.05 versus Ang II.

As described above, Ca^2+^-CaM-CaMK pathway is also involved in the regulation of *CYP11B2* transcription and aldosterone synthesis [32]. Therefore, we examined the effects of PA024 on this pathway. As shown in Fig 8, PA024 increased the Ang II-mediated intracellular Ca^2+^ concentration. From this result, we speculated that the PA024-mediated suppression of *CYP11B2* transcription is not through Ca^2+^-CaM-CaMK pathway and that PA024 may directly suppress NURR1 expression or NURR1 binding to the Ad5 element, resulting in the suppression of *CYP11B2* transcription. Indeed, we demonstrated that the PA024-mediated suppression of NURR1 or *CYP11B2* transcription was mediated via RXRα by RXRα overexpression or knockdown experiments (Fig 5A–5D). Since a half-site of RXRE (AGGTCA) exists on the NURR1 promoter region, PA024 may suppress NURR1 transcription through this region via RXRα, although further studies are needed to confirm it. Moreover, since RXRβ knockdown did not affect the PA024-mediated suppression (data not shown), RXRα may be mainly involved in the effect.

We also demonstrated that PA024 significantly suppressed Ang II-induced mRNA expressions of *StAR*, *HSD3β2* and *CYP21A2*, which are a steroidogenic enzyme group involved in aldosterone biosynthesis in H295R cells (Fig 4A, 4D and 4E). Thus, decrease expressions of these enzymes may also contribute to the PA024-mediated suppression of aldosterone synthesis. Furthermore, recent studies have shown that *HSD3β1* is expressed exclusively in the adrenal zona glomerulosa (ZG) [33,34]. and Ang II could stimulate the expression of *HSD3β*1, although the effects of *HSD3β1* on aldosterone biosynthesis have remained unclear [35]. Therefore, we also examined the effects of PA024 on *HSD3β1* mRNA expressions. After 6 h Ang II treatment, *HSD3β1* mRNA expression dramatically increased up to approximately 10-fold over the basal level. However, we could not observe the response of *HSD3β1* mRNA expression by PA024 treatment (Fig 4C).

Both *CYP11B1* mRNA expression and cortisol secretion in H295R cells were significantly increased by Ang II stimulation (Figs 1F and 4G). Previous studies have shown that treatment with Ang II increased the expression of *CYP11B1* mRNA in addition to that of *CYP11B2* mRNA [36], and cortisol secretion through a mechanism of the Protein Kinase D activation in H295R cells [37]. We here demonstrated that PA024 suppressed the Ang II-induced *CYP11B1* mRNA expression (Fig 4G). In addition, PA024 suppressed the Ang II-induced mRNA expressions of *StAR*, *HSD3β2* and *CYP21A2*, which are also involved in cortisol biosynthesis in H295R cells (Fig 4A, 4D and 4E). However, PA024 did not affect the Ang II-induced cortisol secretion in H295R cells (Fig 1F). Therefore, the use of PA024 may not induce glucocorticoid deficiency, and the clinical value of PA024 might not be limited.

Our previous study showed that PPARγ agonist pioglitazone inhibits *CYP11B2* mRNA expression via Ca^2+^-CaM-CaMK pathway [14]. In this study, we showed that PA024 inhibited the expression of *CYP11B2* through different mechanisms. Therefore, we tested the combination of PA024 and pioglitazone on *CYP11B2* expression. As shown in Fig 6, we demonstrated that co-treatment with 10 μmol/L pioglitazone and 10 μmol/L PA024 significantly suppressed the Ang II-induced *CYP11B2* mRNA expression compared to separate administration. Thus, the combination of PA024 and pioglitazone may cause synergistic suppressive effects on *CYP11B2* mRNA expression via different mechanisms.

We confirmed that PA024 suppressed both SBP and DBP *in vivo* (Fig 7C and 7D). During the course, no significant difference was observed in the BW change between the control group and the PA024 treated group (Fig 7A). On the other hand, PA024 transiently decreased the HR after treatment for 6 weeks (Fig 7B). There were no deaths in THM-administered PA024. These data indicate that PA024 may affect not only the BP but also the HR transiently. RXR has been demonstrated to play an important role in cardiac development. Loss-of-function mutation of the RXRα gene in the mouse germ line resulted in the hypoplastic development of the ventricular chambers of the heart [38]. The conduction system disturbances found in the RXRα homozygous mutant (-/-) embryos may reflect the requirement of the developing conduction system for the RXRα signaling pathway, or it may be secondary to the failure of septal development [39]. Zhu et al. recently reported that RXRα agonist bexarotene treatment attenuated myocardial hypertrophy in spontaneously hypertensive rats by modulating the activation of the liver kinase B1 (LKB1)/AMP-activated protein kinase (AMPK)/p7056 kinase signaling pathway, which occurs independently of the BP [40]. Although the mechanism(s) involved in the changes of lowering heart rate by PA024 are unclear, it is at least certain that RXR has an important role in heart development and may possibly be involved. In this experiment, high-dose bexarotene treatment (100 mg/kg, by oral gavage once daily for 12 weeks) resulted in a slight increase in aspartate aminotransferase, alanine aminotransferase, and blood urea nitrogen [40]. In other animal experiments showing suppression of vascular intimal hyperplasia, HX630 treatment (fed 5 or 10 mg/kg/day for 4 weeks) showed no significant alternations in BW, blood chemistry, or blood count [41]. Therefore, high-dose RXR agonist might be potentially toxic.

In conclusion, we demonstrated that PA024 suppressed Ang II-induced *CYP11B2* mRNA expression and promoter activity in a dose-dependent manner, and aldosterone secretion at its high concentrations in H295R cells. We also found that the decrease of Ad5 activation via NURR1 decrease may contribute to the PA024-mediated suppression of *CYP11B2* transcription. These effects of PA024 were shown to be exerted via RXRα. Furthermore, we confirmed that PA024 treatment lowered both the SBP and the DBP in male THM presenting chronic hypertension *in vivo*. Thus, these results suggest that the RXR pan-agonist PA024 might be a candidate anti-hypertensive drug that acts via the suppression of aldosterone synthesis and secretion.
